# Feedback Recorrection Semantic-Based Image Inpainting Under Semi-Supervised Learning

**DOI:** 10.3390/s25216669

**Published:** 2025-11-01

**Authors:** Xueyi Ye, Ruijie Tan, Mingcong Sui, Huahua Chen, Na Ying

**Affiliations:** Laboratory of Pattern Recognition and Information Security, Hangzhou Dianzi University, Hangzhou 310018, China; xueyiye@hdu.edu.cn (X.Y.); smc13777849137@163.com (M.S.); iseealv@hdu.edu.cn (H.C.); yingna@hdu.edu.cn (N.Y.)

**Keywords:** image inpainting, feedback recorrection, cross-image semantic consistency, semantic segmentation, semi-supervised learning

## Abstract

Image semantics, by revealing rich structural information, provides crucial guidance for image inpainting. However, current semantic-guided inpainting frameworks generally operate unidirectionally, relying on pre-trained segmentation networks without a feedback mechanism to adapt segmentation dynamically during inpainting. To address this limitation, we propose an innovative inpainting methodology that incorporates semantic segmentation feedback recorrection via semi-supervised learning. Specifically, the fundamental concept involves enabling the initial inpainting network to deliver feedback to the semantic segmentation model, which subsequently refines its predictions by leveraging cross-image semantic consistency. The iteratively corrected semantic segmentation maps serve to direct the inpainting neural network toward improved reconstruction quality, fostering a synergistic interaction that enhances both segmentation accuracy and inpainting performance. Furthermore, a semi-supervised learning strategy is implemented to reduce reliance on ground truth labels and improves generalization by utilizing both labeled and unlabeled datasets. We conduct our methodology on the CelebA-HQnd Cityscapes datasets, employing multiple quantitative metrics including LPIPS, PSNR, and SSIM. Results demonstrate that the proposed algorithm surpasses current methodologies: on CelebA-HQ dataset, it achieves a 5.89% reduction in LPIPS and a 0.52% increase in PSNR, with notable improvements in SSIM; on the Cityscapes dataset, LPIPS decreases by 6.15% and SSIM increases by 1.58%. Ablation studies confirm the effectiveness of the feedback recorrection mechanism. This research provides novel insights into synergistic interactions between segmentation and inpainting, demonstrating that fostering such interactions can substantially improve image processing performance.

## 1. Introduction

Recent advancements in image inpainting algorithms have been fueled by the rapid progress of deep learning methodologies. The primary objective of image inpainting is to generate plausible content for missing or corrupted regions that maintains both visual fidelity and semantic coherence. Ideally, the reconstructed images should demonstrate high perceptual quality and semantic consistency. This technology is widely applied across various domains, including occlusion repair, image editing, and object removal. A notable trend in recent research involves integrating combined semantic information, typically guided by semantic segmentation maps, to provide contextual understanding of damaged areas. Leveraging these semantic priors significantly enhances texture synthesis accuracy, ensuring that the repaired regions align seamlessly with the overall semantic structure, thereby optimizing the efficacy of image inpainting.

Although the advantages of semantic guidance in image inpainting have been discussed previously, the primary challenge remains in acquiring precise and comprehensive semantic maps prior to texture synthesis in damaged regions. Consequently, the commonly used strategy is a single guidance method, which adopts a cascade process: initially, semantic prediction is performed on the occluded area to generate a complete semantic map; subsequently, this predicted semantic map directs the texture repair process [1,2,3,4,5]. For instance, Song et al. [2] proposed a two-stage framework wherein the first stage utilizes the damaged semantic map and mask image to predict a complete semantic map, while the second stage employs this predicted semantic map as a guide to complete the filling of missing textures. Xiong et al. [3] introduced an image inpainting model leveraging foreground perception, utilizing the contour information of foreground objects to guide the overall repair process. Yang et al. [4] developed a restoration approach integrating semantic contextual cues with edge feature guidance. Firstly, the semantic structure of the image is reconstructed; then, the reconstruction of the edge contour is guided, culminating in the synthesis of the complete image through the fusion of semantic and edge information. Li et al. [5] proposed a semantic prior-driven inpainting algorithm that integrates high-level semantic features with low-level visual information. By leveraging a semantic attention-aware module to enhance detail representation, their method achieves improved performance in complex background regions. While these single-stage guidance methods offer certain advantages in providing semantic cues, they inherently encounter limitations in precisely inferring comprehensive semantic layouts, particularly in image inpainting scenarios involving extensive missing regions. Moreover, the lack of a feedback mechanism in this unidirectional guidance system implies that errors in the initial semantic prediction will be directly transferred to the succeeding correction process, leading to the accumulation of errors.

To address the aforementioned challenges, the progressive guidance approach employs hierarchical semantic prediction to facilitate dynamic correction [6,7,8,9,10,11,12]. The fundamental architecture of these techniques typically includes creating an iterative optimization mechanism between texture correction and semantic prediction. To direct the inpainting process and increase inpainting accuracy, Liao et al. [7] suggested a single-stage semantic-guided image inpainting model that progressively creates high-resolution semantic maps. Liao et al. [8] achieved the focus optimization of single texture repair guided by semantics by introducing the consistency prior between semantics and texture information in the repair model. Yu et al. [9] suggested an end-to-end multi-modal guided repair network architecture, which consists of two auxiliary branches for semantic segmentation and edge guidance, respectively, and a core repair branch, in light of the high computational complexity brought on by multi-stage implementation. In order to achieve semantic consistency, Chen et al. [10] integrated low-level feature maps as prior information for texture repair and utilized high-level semantic features to direct the repair process of structure-aware features. In order to achieve high-quality picture restoration in complicated scenarios, Zhang et al. [11] developed the semantic pyramid network, which gradually extracts multi-scale semantic priors to refine low-level visual data. Zhang et al. [12] introduced a semantic segmentation-guided image inpainting framework, wherein the feedback loop within the restoration process offers semantic-level guidance, facilitating iterative interaction to progressively enhance the inpainting accuracy.

The progressive guidance strategy predominantly utilizes texture features to predict the semantic map and iteratively update the texture representations in subsequent layers. However, inaccuracies in semantic predictions derived from texture features can result in the reinforcement of semantic discrepancies, thereby compromising the fidelity of the inpainted image. An efficient method for correcting semantic errors is absent from Zhang et al. [12]’s interactive framework, despite the fact that it combines semantic segmentation and image inpainting to improve reconstruction quality. Additionally, a feedback loop that intensifies semantic deviations during repetitive inpainting is induced by the bidirectional dependency between semantic information and texture features, ultimately compromising the coherence and rationality of the final restoration results.

Following a comprehensive analysis of the underlying causes of the issue, this study proposes a semi-supervised semantic feedback recorrection framework. To address potential semantic drift within the guidance mechanism, the approach employs a cross-image semantic consistency constraint to facilitate semantic refinement. Reliable samples are selected for feedback into the semantic segmentation network to enable iterative re-calibration, establishing a closed-loop process of “generation-screening-re-optimization”. Within the semi-supervised learning paradigm, cross-image semantic consistency involves leveraging features across both labeled and unlabeled datasets to ensure intra-class feature coherence while preserving inter-class discriminability. This paradigm of enhancing model robustness to incomplete data by mining intrinsic similarities between data has also been proven effective in other fields, such as few-shot learning [13]. Utilizing the robust feature extraction capabilities of deep neural networks, the proposed methodology effectively captures semantic congruence across images, correcting potential semantic flaws in unlabeled data.

To evaluate the efficacy of the proposed algorithm, we developed a validation framework integrating the interactive optimization of the semantic segmentation network and the image inpainting model, specifically targeting image inpainting tasks. The framework enhances semantic label generation during the inpainting process and employs semi-supervised semantic feedback mechanisms for semantic error correction. Comprehensive experiments were conducted on the CelebA-HQ and Cityscapes datasets, using both qualitative and quantitative assessments to compare our method to existing image inpainting algorithms. The results demonstrate that the algorithm markedly improves inpainting accuracy and visual coherence through the synergistic interaction between the semantic segmentation and image inpainting modules, confirming its applicability in complex damaged scene scenarios. The primary contributions of this study are as follows:

- A progressive inpainting framework is proposed, comprising initial inpainting, semi-supervised semantic feedback recorrection, and refined inpainting stages. The semantic segmentation model tailored for image inpainting is derived from the preliminary inpainting outputs, with semantic correction implemented through cross-image semantic consistency constraints and an iterative interaction mechanism to mitigate error propagation inherent in initial inpainting.
- A semi-supervised, semantic-guided inpainting paradigm is introduced, establishing a teacher–student network architecture supported by adversarial training.
- Theoretically, the study explores the collaborative optimization mechanism between semantic priors and the inpainting model, thereby advancing the foundational research on multimodal information fusion within the domain of image inpainting.

This paper’s remaining sections are arranged as follows: a thorough analysis of the relevant research is given in Section 2, and the mechanism of the proposed semi-supervised semantic feedback recorrection algorithm is thoroughly described in Section 3. The experimental design and data analysis are presented in Section 4, and a summary of the research findings is provided in Section 5.

## 2. Related Work

The relevant research in three sub-domains—traditional picture inpainting, deep learning-based image inpainting, and semantic prior-based image inpainting—will be briefly reviewed in this section.

### 2.1. Traditional Image Inpainting

Traditional digital image inpainting methods can be divided into diffusion-based image inpainting and sample-based image inpainting, both of which use the relationship between pixels to fill the damaged area.

The diffusion-based method draws on the diffusion process of pixels to propagate the information of the known region to the damaged region. The concept of digital image inpainting was first proposed by Bertalmio et al. [14], and they proposed the Bertalmio–Sapiro–Caselles–Ballester (BSCB) model based on a partial differential equation which smoothly propagates the known image information along the direction of the isophote until the damaged area is completely repaired. After that, Bertalmio et al. [15] proposed a joint interpolation method combining image gray level and vector field and improved the BSCB model by introducing light such as hydrodynamic propagation. Shen et al. [16] proposed the Total Variation (TV) model, which uses the minimization energy functional to achieve image completion. Later, it was improved to the Curvature-Driven Diffusions (CDD) model [17] to overcome the limitation that the TV model cannot repair the visual connectivity of the image. However, in addressing extensive regions of data deficiency, diffusion-based inpainting techniques may result in prolonged processing durations or loss of detail fidelity.

Sample-based inpainting techniques typically extract analogous local regions or textures from known image areas to reconstruct missing or damaged regions. The content-aware image inpainting algorithm developed by Criminisi et al. [18] employs contextual image information to guide the inpainting process; however, its similarity measurement function exhibits instability, often resulting in incorrect fill order. To address this issue, researchers have incorporated curvature, gradient information, or pixel color differences into the priority function calculation. Additionally, Barnes et al. [19] introduced an efficient nearest neighbor algorithm aimed at reducing computational complexity.

Traditional image inpainting techniques predominantly depend on the information from known regions, with the reconstructed content in the damaged area primarily involving texture replication, thereby limiting the generation of novel content from the original image data. Subsequently, some researchers have employed external databases for image retrieval to enhance inpainting performance by providing additional contextual information; however, these approaches are characterized by computational inefficiency and susceptibility to matching inaccuracies, resulting in suboptimal restoration quality. The advent of deep learning has revolutionized this field by leveraging its robust learning and inference capabilities, offering innovative solutions for image inpainting. An increasing number of researchers are utilizing deep neural network architectures to overcome the limitations inherent in traditional inpainting methodologies.

### 2.2. Deep Learning-Based Image Inpainting

The Generative Adversarial Network (GAN), introduced by Goodfellow et al. [20] in 2014, represents a seminal architecture within the domain of deep learning. Its robust generative capabilities are instrumental in applications such as image generation, style transfer, and image inpainting. This model underpins the methodology employed in this study. Additionally, the Convolutional Neural Network (CNN) constitutes a pivotal deep learning framework capable of extracting high-level semantic features from images, and it is extensively utilized across various computer vision tasks. Since 2016, the application of GANs in image inpainting research has experienced rapid advancement, notably following the development of the Context Encoders (CE) network by Pathak et al. [21], which integrates autoencoder structures with GANs, thereby pioneering new avenues in deep learning-based image inpainting.

Since then, researchers have implemented multiple architectural optimizations, including global and local inpainting networks [22], multi-branch convolutional structures [23], and gated convolutional layers [24], which have progressively enhanced the structural coherence and detail fidelity of image inpainting. Concurrently, the integration of attention mechanisms has been systematically advanced; Yu et al. [25] introduced the contextual attention module, effectively leveraging long-range dependencies to improve the restoration of complex scenes. Pyramid pooling [26] and circular feature inference [27] further augment the model’s capacity to comprehend texture and semantic information. Beyond network architecture, the incorporation of perceptual loss and style loss functions [28] has contributed to generating reconstructed images with heightened realism and stylistic consistency at the semantic level.

In recent developments, prior information—such as edge detection [29], semantic segmentation [2,30], and related techniques—has been extensively employed to facilitate the restoration of large-scale or complex image defects, thereby significantly improving semantic coherence. Among these [31,32,33,34], semantic priors, as a pivotal guidance mechanism, have demonstrated efficacy in enhancing image inpainting performance. The critical role of semantic information in image inpainting tasks has consequently positioned it as a central focus within digital image inpainting research.

### 2.3. Prior Semantic-Based Image Inpainting

By incorporating high-level semantic information such as object classes and scene structure, the semantic prior-based image inpainting technique significantly enhances the coherence between the inpainting area and global content, addressing the limitations of traditional methods that depend solely on low-level features. Typical implementation strategies encompass semantic segmentation, generative adversarial networks (GANs), and multi-task learning frameworks. The utilization of semantic priors guides the inpainting process toward producing structurally coherent and semantically consistent results, particularly in complex environments or massive defects.

The existing approaches can be classified into two primary categories: single semantic guidance and progressive semantic guidance. Single semantic guidance typically involves reconstructing semantic segmentation maps or structural contours prior to guiding the image inpainting process. For instance, SPG-Net and other methods [2,3,4,5,35,36] improve the coherence of fine details and semantic consistency. However, when confronted with intricate structural complexities and massive defects, these methods often encounter challenges, including insufficient detail and unnatural boundaries.

The progressive semantic guidance process incrementally refines both semantic and texture representations across multiple stages, alternately optimizing structural comprehension and content synthesis to enhance the continuity and precision of inpainting results. Related approaches [7,8,9,10,12,37] typically integrate high-level and low-level features, multi-branch network architectures, or joint segmentation and inpainting optimization strategies, thereby achieving superior performance in restoring large-scale and complex scenes. Nonetheless, the efficacy of the progressive methodology heavily depends on the semantic accuracy established in the initial inpainting stage, since early prediction errors may propagate and degrade the final output. The mechanisms for semantic correction require further refinement to mitigate such issues.

Therefore, this study introduces a semantic feedback-based recorrection methodology that effectively integrates semantic feedback recorrection with the inpainting process, emphasizing the improvement of restoration fidelity and semantic coherence.

## 3. Method

This section details the proposed semi-supervised semantic feedback recorrection algorithm. Initially, the overall system architecture is outlined. Subsequently, the methodology is then detailed, consisting of three phases: initial inpainting, semi-supervised semantic feedback recorrection, and refined inpainting. Finally, the formulation of the associated objective function is presented.

### 3.1. Framework Overview

**Figure 1:**
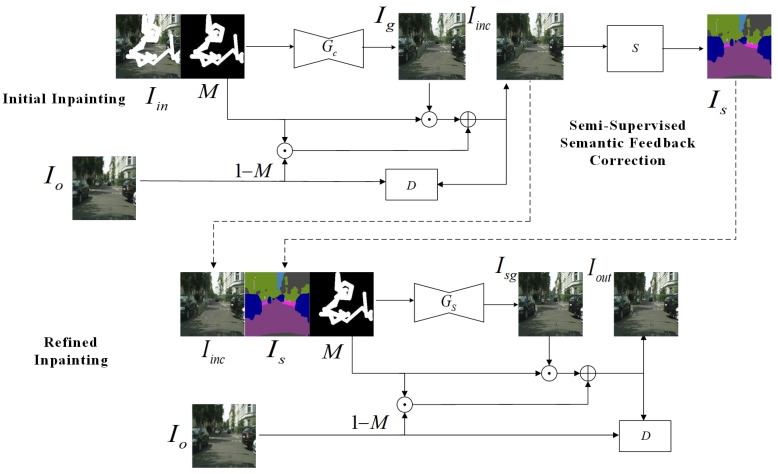
Overall process architecture.

To systematically address the prevalent issues of texture distortion and semantic deviation accumulation in image inpainting, this study introduces a novel three-stage progressive inpainting architecture. The comprehensive workflow is illustrated in Figure 1. The proposed methodology comprises three fundamental phases: initial inpainting, semi-supervised semantic feedback recorrection, and refined inpainting. Each phase is integral to optimizing the overall inpainting efficacy of the algorithm.

Let *M* denote a binary mask, where 1 indicates the missing regions and 0 specifies the known (unmasked) areas, and let $I_{in}$ represent the corrupted image with missing regions. The process commences with the damaged image $I_{in}$ accompanied by a binary mask *M*. The initial inpainting module functions as the fundamental component within the image inpainting pipeline, designed to produce a preliminary structural prediction for the missing regions. We employ an initial generator, denoted as $G_{c}$, which processes the damaged image $I_{in}$ as input and generate a coarse inpainted output $I_{g}$. The final initial inpainting image, $I_{inc}$, is obtained by integrating the content of the generated masked area with the known pixels of the unmasked area in the original image. During this phase, when the texture and intricate details of the inpainted area are complex, the algorithm depends on the model for approximate inpainting. While capable of restoring the overall structural integrity, this approach may introduce semantic inaccuracies or distortions in fine details.

To address the semantic inaccuracies introduced during the initial inpainting process, this research introduces a semi-supervised semantic feedback recorrection module. The module leverages both labeled images and unlabeled images within a semi-supervised learning framework to enhance semantic segmentation of the initially inpainted image. Specifically, the semantic segmentation model, denoted as *S*, performs post-inpainting semantic annotation, identifying and rectifying regions with semantic discrepancies to generate a more precise semantic map, denoted as $I_{s}$. This semi-supervised feedback mechanism effectively exploits latent semantic information within unlabeled images, thereby mitigating the limitations posed by scarce labeled data. Although the schematic in Figure 1 presents a simplified overview of the module, detailed exposition is provided subsequently. The primary advantage of this approach lies in utilizing both labeled and unlabeled data to optimize the semantic correction capability of the segmentation model trained on the initial inpainted output. In scenarios characterized by limited semantic annotations, reliance on unlabeled data for semantic refinement emerges as a crucial and efficacious strategy.

In the final refinement stage of inpainting, the objective is to synthesize a high-fidelity inpainted image, denoted as $I_{\mathrm{out}}$. A refinement generator, denoted as $G_{s}$, is introduced and takes both the initial inpainted image $I_{inc}$ and the corrected semantic map $I_{s}$ as dual inputs. By leveraging the refined semantic guidance, the inpainting process achieves enhanced structural coherence and visual realism, effectively mitigating semantic inconsistencies present in the preliminary inpainting stage and ensuring the output aligns more accurately with real-world scene semantics.

Furthermore, the comprehensive framework training is supervised by an auxiliary discriminator to validate the authenticity of the synthesized images. This coarse-to-fine progressive architecture enhances the structural coherence, semantic plausibility, and perceptual fidelity of the inpainting results.

### 3.2. Initial Inpainting

The primary objective of the initial inpainting module is to perform an initial restoration of the missing regions by generating preliminary pixel content. The process commences with an original image $I_{o}$ and a corresponding binary mask *M* (wherein a pixel value of 1 signifies a corrupted region and 0 indicates an intact region). The masked input image $I_{in}$, which is fed to the generator, is formulated as $I_{in}=I_{o}⊙\left(1-M\right)+M$, where ⊙ denotes element-wise multiplication. The overall initial inpainting module aims to generate a preliminary repaired image $I_{inc}$. This repair process is implemented using a generator $G_{c}$, typically structured as an encoder–decoder architecture. The encoder component of $G_{c}$ extracts features from $I_{in}$, and the decoder component subsequently utilizes these features to reconstruct the pixels of the missing regions, producing the raw generative output $I_{g}=G_{c}\left(I_{in},M\right)$. This generator architecture effectively leverages both global and local features learned from the image context. The output of this generator $I_{g}$ provides the synthesized content for the missing regions. The final preliminary repaired image $I_{inc}$ is then composed by integrating the generated content from $I_{g}$ for the masked regions *M* with the known pixels from the original image $I_{o}$ for the unmasked regions (1−M), typically as $I_{inc}=I_{g}⊙M+I_{o}⊙\left(1-M\right)$. The main task of this initial inpainting stage is to restore the missing pixels and reconstruct basic texture information. While the repairs achieved at this stage may not be flawless, they establish a crucial foundation for the subsequent semi-supervised semantic feedback recorrection and fine-grained refinement processes.

**Figure 2:**
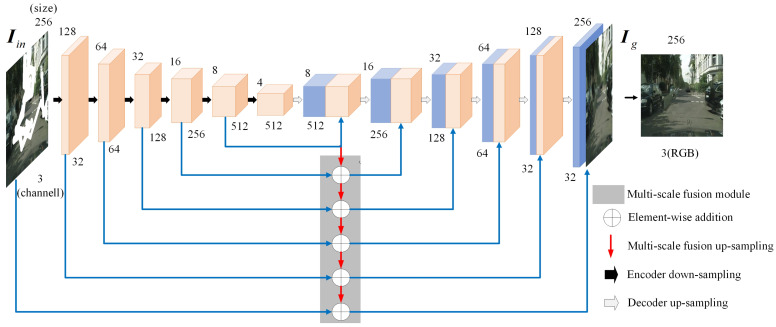
Generator of initial inpainting module.

The architecture of the generator employed in the initial inpainting network is depicted in Figure 2. Its design is inspired by the U-Net architecture [38], which offers notable advantages in feature fusion. Through skip connections, the network effectively transfers high-level feature information, extracted by the encoder, to the corresponding layers in the decoder, thereby facilitating a layer-by-layer fusion of feature maps. This multi-scale, layer-by-layer fusion strategy ensures the efficient propagation and preservation of high-level semantic features, ultimately enhancing the quality and intricate details of the inpainted image. Specifically, the generator comprises an encoder with six down-sampling convolutional layers and a decoder with six up-sampling convolutional layers. Each down-sampling layer employs a 3 × 3 convolution operation with a stride of 2 to reduce the spatial dimensions of the feature map, incorporating batch normalization and the ReLU (rectified linear unit) activation function to improve the model’s ability to capture non-linear relationships. Symmetrically, each upsampling layer in the decoder first utilizes bilinear upsampling to double the spatial resolution of the feature map. This is succeeded by a 3 × 3 convolution, batch normalization, and an activation function to progressively refine the inpainted output.

Within this network architecture, skip connections play a pivotal role. These connections directly transfer low-level feature information from the encoder to corresponding layers in the decoder, thereby effectively preserving the intricate detailed structure of the image. Such a feature transfer mechanism is crucial not only for reconstructing missing regions within the image but also for maintaining the coherence and continuity of the global image structure, supplying a wealth of visual cues.

**Figure 3:**
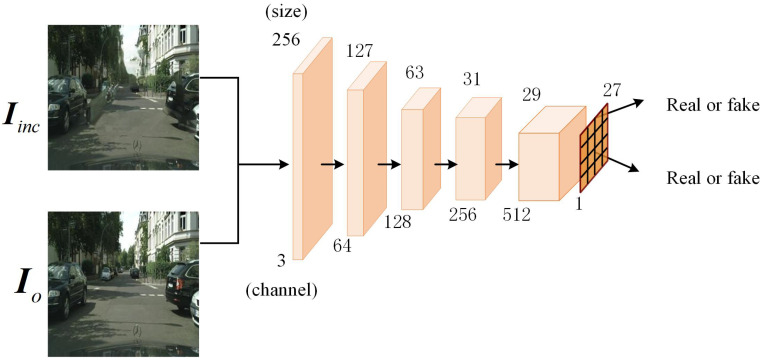
Initial inpainting discriminator network.

Illustrated in Figure 3, the discriminator architecture is adapted from the Markovian discriminator (PatchGAN) [39], effectively meeting the algorithmic requirements for local realism assessment. This discriminator comprises four sequential convolutional layers, each followed by a ReLU (rectified linear unit) activation function. This configuration aims to enhance the network’s capacity for modeling non-linear relationships and improve its overall robustness. Finally, the output layer is coupled with a sigmoid activation function, which yields a probability score indicating the assessed authenticity of the input image patch. The discriminator not only focuses on the global structure of the image but also evaluates local areas (i.e., small patches) of the image, improving the consistency of details in the inpainted regions.

### 3.3. Semi-Supervised Semantic Feedback Recorrection

**Figure 4:**
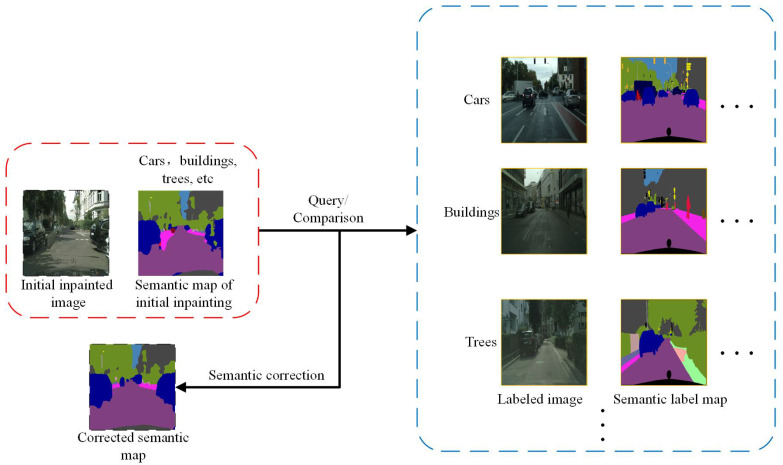
Conceptual representation of semantic consistency across images.

This section introduces a semi-supervised semantic feedback recorrection methodology rooted in cross-image semantic consistency, which is applicable to both semantic segmentation and image inpainting tasks. Within the domain of semantic segmentation, the principle of cross-image semantic consistency involves leveraging the reliable and accurate semantic information from labeled images to correct mislabels in unlabeled images. In image inpainting, for images that have undergone initial inpainting and may still exhibit semantic errors or artifacts, the objective is to enhance the semantic segmentation quality of these initially inpainted images, correct semantic inaccuracies introduced during this initial inpainting process, and reduce the discrepancy between ground-truth semantic labels and those of the initially inpainted results. This is achieved by utilizing the semantic relationship between the initially inpainted image and the real image (including both the true and pseudo-labels). The concept of cross-image semantic consistency in the image inpainting task is illustrated using an example of an urban street view, as shown in Figure 4.

As illustrated in Figure 4, the corrected semantic map derived from the initial inpainting shows that semantic correction for the car (blue) and the sidewalks (pink) on both sides of the road align well with the expected outcomes. This indicates that it is feasible to use relatively reliable and accurate semantic information to correct the semantic errors present in the initial inpainting process. For instance, consider an initial inpainting image featuring a car with uncertain or ambiguous regions. An image selection method is employed to retrieve labeled images of the same category (i.e., “car”) along with their corresponding semantic labels. Subsequently, semantic correction is performed by computing the similarity between deep features extracted from the initial inpainting and those from the selected labeled image.

However, selecting reliable labeled images remains a challenge. This image selection process can be framed as a ranking problem. Traditional image selection tasks tend to rank based on quality metrics, while this work adopts a cross-image semantic consistency approach for image selection. The core principle of this image selection method is to rank images based on the estimated similarity between the semantic categories of each image, ensuring that reliable pseudo-labels are selected during the inpainting process, thereby providing robust support for the semi-supervised learning process. The specific steps for selecting images to serve as reliable pseudo-labels are as follows:

1. Compute Category Anchor Vectors: Features for each semantic category are extracted from labeled images using a pre-trained semantic segmentation model. Subsequently, the average feature for each semantic category across all relevant images is computed to generate a category anchor vector, representing the prototypical appearance of that category within the labeled dataset.
2. Compute Similarity: The similarity between the category features of an input image and the pre-computed category anchor vectors is determined. Cosine similarity is employed to measure the closeness of the input image’s features to the anchor vectors of various categories. This process yields a category-wise feature map (or similarity scores) that reflects the semantic relationship between the input image and the established category prototypes.
3. Obtain Semantic Segmentation Masks: The pre-trained semantic segmentation model is utilized to segment the input image, generating per-pixel category predictions, referred to as category masks (i.e., the predicted semantic label for each pixel).
4. Define Similarity Distance: A similarity distance is defined by computing the cross-entropy loss between the category-wise feature map (from step 2) and the obtained category masks (from step 3). This quantifies the discrepancy between the feature-based similarity to category prototypes and the direct pixel-level semantic predictions. Smaller values indicate higher similarity.
5. Image Ranking and Selection: Input images are ranked based on the computed similarity distance. A higher rank (corresponding to a smaller similarity distance) indicates that the pseudo-labels derived from the input image are deemed more reliable and are thus selected for subsequent processing.

In the context of semi-supervised semantic segmentation, unlabeled images are partitioned into reliable and unreliable sets, to which a phased training strategy is applied. This process effectively establishes a teacher-student architecture for the segmentation models. Here, a model from an earlier phase (e.g., from Step 1 or 4) acts as the teacher, generating pseudo-labels or providing initialization. The model in the subsequent phase (e.g., in Step 4 or 5) then acts as the student, which learns from these outputs and is refined on an expanded dataset that includes the filtered reliable set. The core of this strategy is to first train the model comprehensively on the reliable set and then leverage the updated student model to optimize predictions on the unreliable set, thereby iteratively enhancing the overall segmentation accuracy. This entire iterative refinement cycle is supported by the adversarially trained inpainting networks, which supply high-quality training images, ultimately boosting the segmentation model’s robustness for the inpainting task.

**Figure 5:**
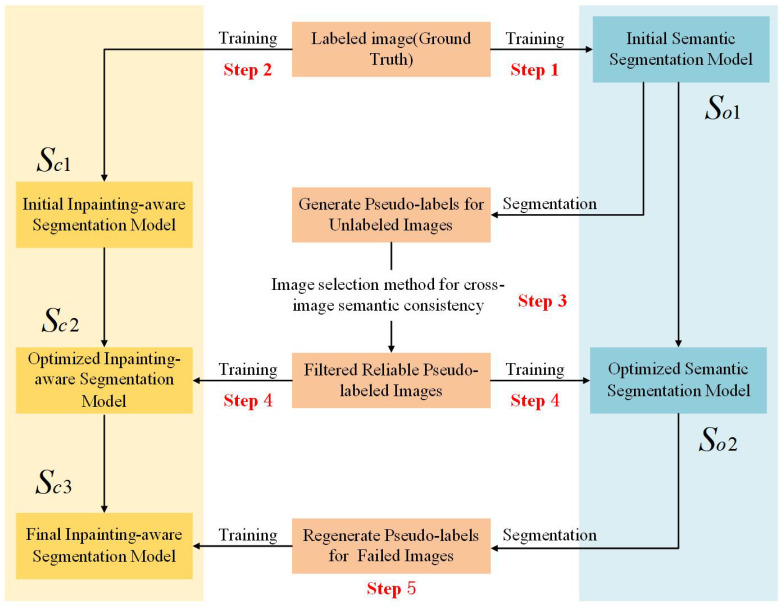
Semi-supervised learning for semantic segmentation training process.

The semi-supervised semantic segmentation network employed in this research builds upon the work of Wu et al. [40]. The implementation pipeline of the semi-supervised semantic segmentation approach, as adopted by our research team, is illustrated in Figure 5. Initially, the model is trained on labeled data to establish the baseline semantic segmentation network and generate initial pseudo-labels for unlabeled data. Subsequently, an image reliability evaluation mechanism is employed to select reliable pseudo-labels from these initial predictions for further training. Through refining predictions on both the reliable set and the unreliable set, the model’s capability to accurately segment previously challenging images is enhanced, ultimately boosting overall segmentation performance.

1. Supervised Initial Training
  Step 1: An initial semantic segmentation model is trained using an image set annotated with ground-truth semantic labels.
  Step 2: Random masks are applied to the same labeled image set. The initial inpainting network then processes these masked images to generate preliminary inpainting results. Subsequently, these inpainted results, along with their corresponding ground-truth semantic labels, are utilized to train an initial inpainting-aware segmentation model.
2. Generation and Filtering of Pseudo-labels
  Step 3: For the unlabeled image set, pseudo-labels are generated by employing the initial semantic segmentation model (derived from Step 1). Subsequently, a cross-image semantic consistency filtering method is applied to select a reliable subset from these generated pseudo-labels.
3. Semi Supervised Iterative Optimization
  Step 4: The initial semantic segmentation model and the inpainting-aware segmentation model are then retrained. This retraining utilizes a combined dataset comprising the original ground-truth labeled images and the previously filtered reliable pseudo-labeled images. This process yields optimized versions of both the semantic segmentation and the inpainting-aware segmentation models.
  Step 5: Subsequently, the optimized semantic segmentation model (from Step 4) is employed to regenerate pseudo-labels for unlabeled images with initial pseudo-labels that did not pass the validation criteria in Step 3. Finally, the inpainting-aware segmentation model undergoes further retraining, this time utilizing the ground-truth labeled images in conjunction with all currently available pseudo-labeled images.

The proportion of ground-truth labeled images utilized within our semi-supervised semantic feedback recorrection framework is a critical determinant of final image inpainting performance. To investigate this relationship, we conducted a comparative analysis on the Cityscapes dataset, varying the ratio of ground-truth labeled images to 1/8, 1/4, and 1/2 of the available supervised training data. The detailed quantitative results are presented in Table 1.

As demonstrated in Table 1, a clear trend emerges: inpainting performance, gauged by PSNR and SSIM, significantly improves across all tested mask ratios with an increasing proportion of ground-truth labels. For instance, under conditions where masks cover 0–20% of the image, the PSNR value increases from 30.23 dB to 32.03 dB as the ground-truth label proportion is raised from 1/8 to 1/2. This observation suggests that with greater availability of authentic data, the model more effectively leverages these reliable annotations, highlighting the substantial positive impact of the ground-truth label quantity on the inpainting outcome. This enhancement is particularly pronounced for images with high defect rates, such as those with mask coverage between 40–60%. In these challenging scenarios, augmenting the proportion of ground-truth labels yields considerable performance gains; specifically, the PSNR improves from 21.48 dB with a 1/8 label proportion to 23.31 dB with a 1/2 label proportion for masks in the 0.4-0.6 range.

Interestingly, Table 1 also reveals that even with a ground-truth label proportion as low as 1/8, the model achieves satisfactory PSNR and SSIM scores (average PSNR of 25.23 dB and SSIM of 0.860). This implies that for less complex inpainting scenarios or where resources are limited, a reduced share of ground-truth data might still meet fundamental training objectives and yield acceptable performance.

Furthermore, the LPIPS scores (where lower is better, indicating higher perceptual similarity) consistently decrease as the proportion of ground-truth labels increases. For instance, the average LPIPS drops from 0.096 at 1/8 to 0.061 at 1/2. This signifies a diminishing perceptual gap between the inpainted outputs and their corresponding ground-truth images, corroborating the quantitative improvements observed with PSNR and SSIM.

These findings indicate that the model achieves optimal inpainting performance when the proportion of ground-truth labels is set to 1/2. Consequently, for all subsequent comparative experiments against other algorithms, this 1/2 proportion of ground-truth labels is adopted as the standard configuration.

The algorithm synergistically integrates image inpainting and semantic segmentation, employing the cross-image semantic consistency method to bolster the semantic understanding of inpainted regions. This improves the accuracy of semantic segmentation, which in turn elevates the quality of image inpainting results. By capitalizing on the strengths of semi-supervised learning, the algorithm not only effectively refines the erroneous labels of unlabeled images but also enhances the semantic accuracy of the repaired images. This approach provides a novel solution for correcting semantic errors in image inpainting tasks.

### 3.4. Refined Inpainting

The primary objective of the refined inpainting stage is to utilize the semantically corrected semantic map $I_{s}$ generated in the previous stage as contextual guidance, in conjunction with the initial inpainted image $I_{inc}$, to produce a visually coherent and highly realistic inpainting result. To achieve this, a refinement generator $G_{s}$ is designed, which accepts as inputs the initial inpainted image $I_{inc}$, the corrected semantic map $I_{s}$, and the binary mask *M* to generate a preliminary refined output: $I_{sg}=G_{s}\left(I_{inc},I_{s},M\right)$, where $I_{sg}$ denotes the initial output of the refinement generator. To restrict updates exclusively to pixels within the masked (unknown) regions, the final inpainted image $I_{out}$ is computed as $I_{out}=I_{sg}⊙M+I_{o}⊙\left(1-M\right)$. where ⊙ signifies element-wise multiplication, and (1−M) indicates the unmasked (known) regions. This operation ensures preservation of the known pixels from $I_{inc}$ while integrating the refined content into the missing areas.

**Figure 6:**
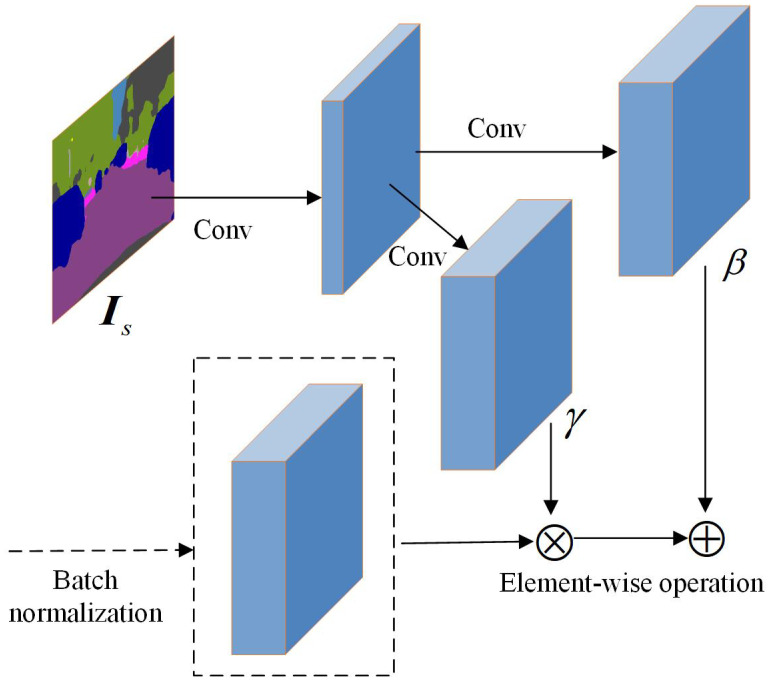
Schematic diagram of SPADE module.

To facilitate effective semantic guidance from $I_{s}$, the semantic propagation modules within the refinement network are extended. Specifically, SPADE (spatially adaptive denormalization) semantic propagation modules are integrated into each skip connections of the generator, enabling spatially adaptive feature modulation conditioned on the semantic map. As illustrated in Figure 6, the SPADE module takes two inputs: the semantic segmentation map and the intermediate feature map from the generator network. Internally, SPADE first processes this semantic segmentation map through a convolutional network, transforming it into an embedding space that matches the spatial dimensions of the generator’s feature map. Subsequently, from this embedding, two separate convolutional layers (constituting a linear transformation) predict spatially adaptive scaling factors γ and offset parameters β for each location. These predicted γ and β parameters are then element-wise applied to modulate the normalized intermediate feature map from the generator, effectively performing a conditional denormalization. SPADE dynamically adjusts the feature map of the generative network based on the semantic information at each spatial location, allowing the synthesized image details to better align with the input semantic labels. Consequently, by incorporating spatial information from the semantic segmentation map, the SPADE module facilitates the dynamic adjustment of feature modulation parameters according to the semantic category of each pixel, thereby enhancing the semantic consistency of the generative model and the perceptual quality of the synthesized image.

**Figure 7:**
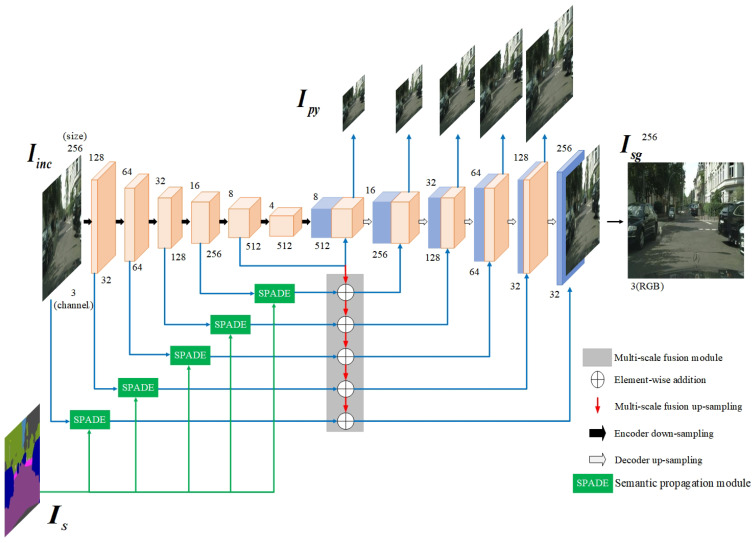
Generator of refined inpainting.

The architecture of the refined inpainting generator is illustrated in Figure 7. Embedding the SPADE module within skip connections ensures that features extracted at various levels of the network maintain semantic consistency. This design facilitates a more reasonable and coherent semantic distribution in the generated image, effectively propagates and modulates semantic information across hierarchical levels, and mitigates information loss during feature transmission. Consequently, this architectural choice enhances the semantic consistency of the inpainted image and optimizes the overall image inpainting performance. The discriminator architecture remains identical to that of the initial inpainting module.

### 3.5. Loss Function

#### 3.5.1. Initial Inpainting

As previously outlined, the primary objective of the initial inpainting phase is to direct the generator to produce a preliminary inpainted image that maintains structural integrity and accurately reconstructs the missing regions. To accomplish this dual aim—ensuring pixel-level fidelity while enhancing perceptual realism—the loss function we devised integrates both global reconstruction loss and adversarial loss, with both components collaboratively guiding the discriminator’s training process. While the global loss primarily ensures content consistency for the generator, both losses contribute to the overall optimization, with the adversarial loss defining the competitive interplay between the generator and discriminator. This global loss component quantifies the pixel-level differences between the generated and ground-truth images, thereby guiding the generator to preserve overarching structures, ensure content accuracy, and meticulously restore image details. Conversely, the adversarial loss enhances the fidelity of the generated image through an adversarial game between the generator and discriminator, enabling the generator to produce high-quality images that are difficult for the discriminator to distinguish as fake. The total loss for the generator, denoted as $L_{G_{c}}$, is defined as a weighted sum of an adversarial loss and a reconstruction loss. The superscript *c* is used throughout to denote components related to this coarse stage. The loss is formulated as:

(1) $$L_{G_{c}}=\lambda_{adv}^{c}L_{adv}^{c}+\lambda_{L1}^{c}L_{L1}^{c},$$

Here, $L_{adv}^{c}$ represents the adversarial loss, which encourages the generation of visually realistic images. The term $L_{L1}^{c}$ is the L1 reconstruction loss, enforcing pixel-level similarity between the generated image and the ground truth. The terms $\lambda_{adv}^{c}$ and $\lambda_{L1}^{c}$ are scalar hyperparameters that balance the contribution of each loss component. The L1 loss $L_{L1}^{c}$ is defined as the L1 norm of the difference between the ground-truth image $I_{o}$ and the inpainted output image $I_{inc}$:

(2) $$L_{L1}^{c}={∥I_{o}-I_{inc}∥}_{1},$$

The generator’s adversarial loss $L_{adv}^{c}$ is a core component of the adversarial training process. It drives the generator to produce samples that are difficult for the discriminator *D* to distinguish from real ones. The primary goal for the generator, with respect to its adversarial loss $L_{adv}^{c}$ is to minimize the perceived distributional difference (as determined by the discriminator) between its generated samples and real samples. This encourages the generator to produce increasingly realistic outputs. In this study, the generator’s adversarial loss $L_{adv}^{c}$ is formulated using the Hinge loss function as follows:

(3) $$L_{\mathrm{adv}}^{C}=-E_{I_{\mathrm{inc}}∼P_{g(I_{\mathrm{inc}})}}\left[D(I_{\mathrm{inc}})\right],$$

In this formulation, $P_{g(I_{inc})}$ denotes the distribution of the generated inpainted image $I_{inc}$, and the expectation E is taken over all samples from this distribution.

The discriminator *D* of the initial inpainting module, as illustrated in Figure 3, is designed to distinguish between real images $I_{o}$ and the repaired images $I_{inc}$ produced by the generator. The total loss of the discriminator $L_{D}$ is given by

(4) $$L_{D}=E_{I_{o}∼P_{data(I_{o})}}\left[\mathrm{ReLU}(1-D\left(I_{o}\right))\right]+E_{I_{inc}∼P_{g(I_{inc})}}\left[\mathrm{ReLU}(1+D\left(I_{inc}\right))\right],$$

Here, the first term calculates the expectation over real images $I_{o}$ sampled from the true data distribution $P_{data(I_{o})}$, while the second term calculates the expectation over generated images $I_{inc}$ from the generator’s distribution $P_{g(I_{inc})}$. The function ReLU(x)=max(0,x) is the Rectified Linear Unit. This Hinge Loss formulation for $L_{D}$ implies that for the discriminator, a real sample $I_{o}$ contributes to the loss only if its score $D(I_{o})<1$ and a repaired sample $I_{inc}$ contributes only if its score $D(I_{inc})>-1$. The use of hinge loss is known to contribute to more stable training and can help accelerate convergence in adversarial settings.

#### 3.5.2. Refined Inpainting

To optimize the refinement network effectively and ensure that the final inpainted output $I_{out}$ attains a high degree of fidelity to the original image $I_{o}$ in terms of pixel accuracy, feature consistency, structural integrity, and perceptual realism across multiple hierarchical levels, we propose a multi-task joint loss function $L_{G_{s}}$. This loss function is explicitly designed to provide comprehensive supervision during the training process of the refinement generator. Specifically, the composite loss for the generator $L_{G_{s}}$ comprises a generative adversarial loss $L_{\mathrm{adv}}^{S}$, a pyramid loss $L_{\mathrm{py}}^{S}$, a perceptual loss $L_{\mathrm{pe}}^{S}$, a style loss $L_{\mathrm{sy}}^{S}$, and an L1 reconstruction loss (global loss) $L_{L1}^{S}$.

(5) $$L_{G_{s}}=\lambda_{\mathrm{adv}}^{S}L_{\mathrm{adv}}^{S}+\lambda_{\mathrm{py}}^{S}L_{\mathrm{py}}^{S}+\lambda_{\mathrm{pe}}^{S}L_{\mathrm{pe}}^{S}+\lambda_{\mathrm{sy}}^{S}L_{\mathrm{sy}}^{S}+\lambda_{L1}^{S}L_{L1}^{S},$$

where $\lambda_{\mathrm{adv}}^{S}$, $\lambda_{\mathrm{py}}^{S}$, $\lambda_{\mathrm{pe}}^{S}$, $\lambda_{\mathrm{sy}}^{S}$ and $\lambda_{L1}^{S}$ are hyperparameters controlling the weight of each component. Each loss term targets a specific aspect of image quality. The formulation and principles of the adversarial loss $L_{\mathrm{adv}}^{S}$ and the L1 reconstruction loss $L_{L1}^{S}$ are analogous to those employed in the initial inpainting module.

The proposed algorithm incorporates a multi-scale fusion strategy to progressively enhance feature propagation across different scales, enabling the generation of inpainted outputs at multiple scales. The pyramid loss $L_{\mathrm{py}}^{S}$ is computed by calculating the L1 distance between features of the predicted outputs and the ground truth at corresponding scales. This multi-scale supervision progressively refines the inpainting of missing regions at each level of the feature pyramid, as formulated in Equation (6):

(6) $$L_{\mathrm{py}}^{S}=\sum_{l}{∥I_{o}^{l}-I_{\mathrm{py}}^{l}∥}_{1},$$

In this formulation, $I_{\mathrm{py}}^{l}$ denotes the inpainted output at scale *l* from the intermediate layers of the generator (refer to the $I_{\mathrm{py}}$ outputs in Figure 7), and $I_{o}^{l}$ represents the ground truth image $I_{o}$ down-sampled to the corresponding scale *l*. The supervision provided by $L_{\mathrm{py}}^{S}$ on the multi-scale fusion process helps to stabilize training and improve the quality of features learned by the decoder at various scales.

To quantify the perceptual discrepancies between the generated and ground truth images, our refined inpainting stage incorporates a perceptual loss. The objective of this loss is to minimize the feature-space distance between the generated image and the ground truth, while penalizing outputs that exhibit significant perceptual deviations from the target. The expectation is taken over the training data distribution, and the loss is calculated by summing the L1-norm differences between feature maps extracted from a pre-trained VGG-19 network:

(7) $$L_{\mathrm{pe}}^{S}=E\left[\sum_{i}\frac{1}{N_{i}}{∥\Phi_{i}\left(I_{o}\right)-\Phi_{i}\left(I_{\mathrm{out}}\right)∥}_{1}\right],$$

Here, $\Phi_{i}\left(\cdot \right)$ denotes the feature map extracted from the *i*-th selected layer of a pre-trained VGG-19 network, and $N_{i}$ is the number of elements in that feature map. Both perceptual and style losses are computed based on these extracted feature maps. The style loss $L_{\mathrm{sy}}^{S}$ aims to quantify discrepancies in visual style, primarily textures and patterns, between the generated image and the ground truth. Given a feature map from layer *j* with dimensions $C_{j}\times H_{j}\times W_{j}$, the style loss $L_{\mathrm{sy}}^{S}$ is computed as the L1-norm distance between the Gram matrices of the VGG-19 feature maps. The expectation is taken over the data distribution and across different feature layers indexed by *j*, as formulated in Equation (8):

(8) $$L_{\mathrm{sy}}^{S}=E_{j}\left[{∥G_{j}^{\Phi }\left(I_{o}\right)-G_{j}^{\Phi }\left(I_{\mathrm{out}}\right)∥}_{1}\right],$$

where $G_{j}^{\Phi }\left(I_{o}\right)$ is the $C_{j}\times C_{j}$ Gram matrix constructed from the feature map, and the same is $G_{j}^{\Phi }\left(I_{\mathrm{out}}\right)$. The application of style loss helps to mitigate the visual artifacts introduced by transpose convolution, ensuring that the repaired images appear more visually appealing and realistic.

## 4. Experimental Results and Analysis

This section initially delineates the experimental settings, encompassing datasets, implementation details, and evaluation metrics. Subsequently, a comparative analysis of the proposed algorithm’s performance against established benchmarks is presented. The contribution of critical modules to overall system efficacy is then examined. Finally, the discussion addresses experimental failure cases and explores their underlying causes.

### 4.1. Experimental Setup

#### 4.1.1. Datasets

For a fair comparison with existing image inpainting methods, all experiments in this paper were conducted using images resized to 256 × 256 pixels for both training and testing. The CelebA-HQ face dataset, which contains semantic segmentation labels, and the Cityscapes urban landscape dataset are utilized. The CelebA-HQ face dataset includes 29,000 training images and 1000 test images, with fine-grained annotations for 19 semantic categories. From the original semantic maps of CelebA-HQ dataset, we merged bilateral components (e.g., left and right eyes) into single categories, reducing the total number of semantic classes to 15 for our experiments. The Cityscapes dataset, encompassing 20 categories of urban street scenes, was also employed. For our experiments on this dataset, the training set was constructed by combining 2975 images from its official training split with 1525 images from its official test split. The 500 images from the official validation split served as our designated test set. This particular partitioning strategy was adopted to leverage the unlabeled images within the official Cityscapes test set, a requisite for our semi-supervised semantic feedback recorrection methodology. Furthermore, this configuration aligns with the experimental setups of several benchmark methods, thereby ensuring a consistent and equitable comparative analysis. Binary masks were employed to delineate the missing regions within the images. Following the methodology proposed by Yu et al. [40], we generated irregular masks for the training dataset, categorized by mask-to-image area ratios into three ranges: 0–20%, 20–40%, and 40–60%.

#### 4.1.2. Implementation Details

To accurately evaluate the performance of the image inpainting algorithm proposed in this paper, both qualitative visual evaluations and quantitative metrics are employed. To ensure the reliability and fairness of the experimental results, we use the deep learning framework PyTorch for training and testing the model, with computations performed on an NVIDIA GeForce RTX 3090 Ti graphics processing unit (GPU). Training was performed with a batch size of 24, and the Adam optimizer was utilized for the optimization of the objective function.

The experiment adopts a multi-loss function training strategy, adjusting the weights of these loss functions based on predefined benchmark values. For the initial (coarse) inpainting stage, the adversarial loss coefficient $\lambda_{\mathrm{adv}}^{C}$ was assigned a value of 1.0, while the L1 reconstruction loss coefficient $\lambda_{L1}^{C}$ was set to 10.0. The semi-supervised learning approach for semantic segmentation follows the methodology proposed by Wu et al. [40]. Additionally, the weights for the loss function of the refined inpainting module are represented as $\lambda_{\mathrm{py}}^{S}=\lambda_{L1}^{S}=1.0$, $\lambda_{\mathrm{adv}}^{S}=\lambda_{\mathrm{pe}}^{S}=0.1$, and $\lambda_{\mathrm{sy}}^{S}=250.0$.

#### 4.1.3. Evaluation Metrics

We utilize several standardized metrics for assessing image inpainting performance, including peak signal-to-noise ratio (PSNR) [41], structural similarity index measure (SSIM) [41], and learned perceptual image patch similarity (LPIPS) [42].

PSNR quantifies the fidelity of the reconstructed image by calculating the mean squared error between the original and inpainted images; higher PSNR values indicate superior inpainting quality.

SSIM evaluates the perceptual similarity between the original and reconstructed images based on luminance, contrast, and structural information, aligning with human visual perception; values approaching 1 denote higher similarity.

LPIPS employs features extracted from a pre-trained deep neural network to measure perceptual differences between image patches, providing an assessment that closely mimics human subjective visual evaluation of perceptual fidelity.

### 4.2. Performance Comparisons

#### 4.2.1. Qualitative Comparison

**Figure 8:**
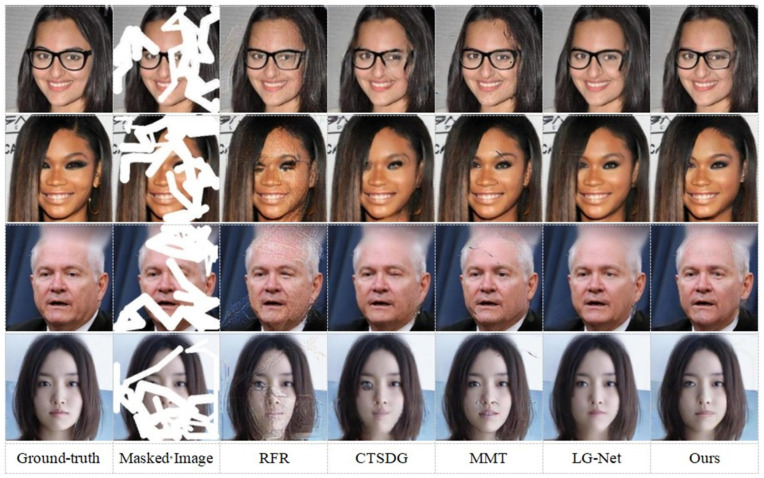
Qualitative comparison on the CelebA-HQ dataset.

On the CelebA-HQ dataset, our proposed algorithm was qualitatively compared against several state-of-the-art methods, including RFR (recurrent feature reasoning) [27], CTSDG (conditional texture and structure dual generation) [34], MMT (multi-modality technology) [9], and LG-Net (local and global network) [43]. Visual comparison results for these methods on the CelebA-HQ dataset are presented in Figure 8. As illustrated in the first row of Figure 8, when inpainting the missing glasses region, most competing methods exhibit noticeable artifacts. For instance, while MMT attempts to incorporate semantic information and edge features, it tends to generate reconstructions marred by significant noise. In contrast, our proposed algorithm achieves a more coherent and artifact-free result in this scenario. In the second row, LG-Net shows errors where the eyebrows do not align with the underlying texture, while MMT again suffers from noise issues. The overall inpainting results of the CTSDG and RFR algorithms are also unsatisfactory for this image. Examining the third row, particularly in the bottom right corner at the face–clothing junction, semantic ambiguities are apparent in the results of both LG-Net and CTSDG. Similarly, the RFR and MMT algorithms display evident inconsistencies between the inpainted and original regions. In the fourth row, semantic ambiguities persist in the outputs of RFR, CTSDG, and LG-Net, which also demonstrate suboptimal inpainting quality, especially concerning the details at the face–neck junction. Overall, the qualitative comparisons indicate that our proposed algorithm surpasses the aforementioned methods in terms of both visual fidelity and semantic coherence. This suggests its enhanced effectiveness for image inpainting tasks, particularly those involving complex scenes with diverse semantic regions.

Streetscape images from the Cityscapes dataset are complex in structure and exhibit significant diversity, thereby posing substantial challenges for image inpainting methodologies. Against this backdrop, the proposed method is qualitatively compared with RFR, CTSDG, MMT, and LG-Net.

**Figure 9:**
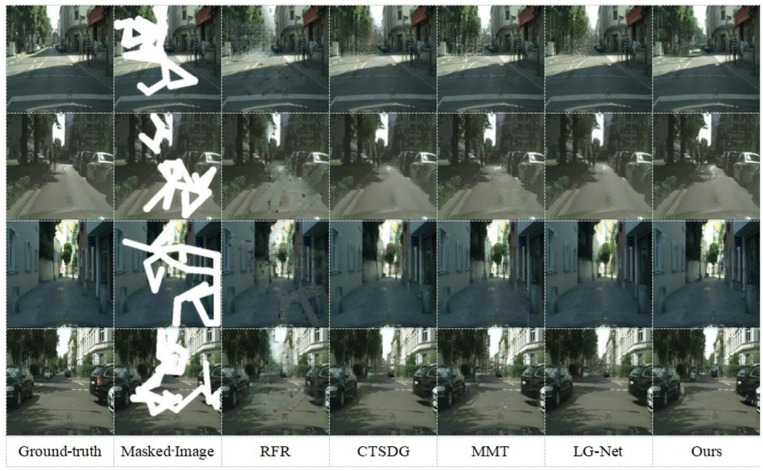
Qualitative comparison on the Cityscapes dataset.

Figure 9 illustrates the visual comparison results of this algorithm and other image inpainting algorithms on the Cityscapes dataset. Compared to the other algorithms, this method demonstrates superior performance in terms of the consistency of the inpainted regions and the overall visual quality. For instance, considering the central perspective regions in the first and fourth rows, the images generated by our algorithm demonstrate discernible advantages in terms of boundary delineation, structural coherence, and visual plausibility. Furthermore, in the third row, our algorithm adeptly reconstructs the junction between the wall and the road. Overall, our method yields inpainted images that are perceptually more natural and visually realistic, particularly excelling in the reconstruction of intricate backgrounds and fine-grained details.

#### 4.2.2. Quantitative Comparison

Following the qualitative assessments, we conducted a rigorous quantitative evaluation of our proposed algorithm’s inpainting performance using the CelebA-HQ dataset to provide objective metrics. For this evaluation, experimental samples were generated by selecting ground truth images and applying irregular defect masks; these masked images then served as inputs for the inpainting models. The inpainting performance of our method was subsequently compared against other relevant approaches through quantitative metrics. This paper employs widely recognized evaluation metrics in image inpainting research, including learned perceptual image patch similarity (LPIPS), peak signal-to-noise ratio (PSNR), and structural similarity index measure (SSIM) [41]. LPIPS assesses perceptual-level visual similarity, whereas PSNR and SSIM quantify low-level pixel-wise fidelity between the ground truth and the inpainted images. Collectively, these metrics offer a comprehensive framework for quantifying the inpainting efficacy of the evaluated methods in this study. The quantitative inpainting results for all compared methods are summarized in Table 2. Data for the comparative methods RFR, CTSDG, LG-Net, W-Net (W-shaped network) [11], and MDTG (mutual dual-task generator) [12] were sourced from the experimental results reported by Zhang et al. [12]. In contrast, the MMT results were obtained through our own reproduction of the method.

As shown in Table 2, our method demonstrates excellent overall performance on the CelebA-HQ dataset. Specifically, it achieves leading results across all metrics at low mask ratios. Meanwhile, under 40–60% high mask ratios, while maintaining superior LPIPS, its PSNR slightly trails LG-Net, and its SSIM ranks second-best. This observation illuminates the inherent limitations of semantic guidance mechanisms when handling extensively damaged regions.

In the quantitative evaluation on the CelebA-HQ dataset (Table 2), our method shows slightly lower PSNR than LG-Net and lower SSIM than MDTG under 40–60% high mask ratios. This observation reveals the limitations of semantic guidance mechanisms when handling extensively damaged regions.

We attribute this performance gap to three key factors. First, as a facial image dataset, CelebA-HQ possesses relatively straightforward semantic hierarchies. Under extreme masking conditions where large homogeneous facial regions are removed, the effectiveness of semantic guidance diminishes considerably, shifting the inpainting priority towards pixel-level texture reconstruction. In such scenarios, architectures like LG-Net, specializing in texture synthesis, demonstrate advantages in achieving pixel-level precision. Second, MDTG exhibits unique advantages in maintaining structural similarity through its specific multi-task learning framework. The generator design of this method may be more focused on preserving the overall structural integrity of images, which is directly reflected in its SSIM advantage. Third, the efficacy of our core innovation—the semantic feedback recorrection mechanism—is inherently dependent on the quality of initial inpainting results. Severe performance degradation during the initial inpainting stage under extreme occlusion directly compromises the reliability of subsequent semantic correction, creating a performance bottleneck.

It is particularly noteworthy that our method achieves a superior LPIPS score of 0.053 within this mask range, demonstrating a significant reduction over all competing methods. Specifically, it achieves a reduction in LPIPS of approximately 1.9% compared to the strongest competitor on this metric, MDTG. The reduction is more substantial when compared to LG-Net and CTSDG, reaching approximately 17.2% and 27.4%, respectively. This evidence confirms that although our method trails in some objective metrics, our approach maintains a decisive advantage in generating visually coherent and semantically plausible results. Considering the comprehensive evaluation across all mask ratios, our method achieves optimal average performance on all metrics, validating the overall effectiveness of our semantic-guided framework.

To address these limitations, future research will focus on enhancing the robustness of the initial inpainting network against extreme occlusion while exploring more resilient semantic reasoning mechanisms—such as multi-scale semantic fusion—that demonstrate greater tolerance to initial inpainting imperfections.

To evaluate performance on the Cityscapes dataset, we similarly employed SSIM, PSNR, and LPIPS as quantitative metrics, with the results summarized in Table 3. The comparative data for RFR, CTSDG, LG-Net, W-Net (W-shaped network) and MDTG (mutual dual-task generator) were all sourced from the experimental findings of Zhang et al. [12]. As with the CelebA-HQ experiments, the MMT results were obtained through our own experimental reproduction.

Analysis of Table 3 reveals that for higher mask ratios (40–60%), our method’s PSNR (23.31 dB) is indeed lower than some specialized methods like CTSDG (24.28 dB). Delving deeper into this phenomenon, we attribute this to the distinct technical focus of different methods: while our semantic feedback recorrection provides advantages in maintaining semantic consistency, the substantial loss of fine-grained details in heavily masked regions presents challenges for complete restoration, consequently affecting pixel-level fidelity metrics like PSNR. This observation aligns with the core trade-off of our method, as also discussed regarding CelebA-HQ dataset. However, a critical finding emerges: within this range, our LPIPS score (0.098) demonstrates clear superiority—showing approximately 30% and 25.2% reductions compared to CTSDG (0.140) and LG-Net (0.131), respectively. This robustly validates the effectiveness of semantic guidance in enhancing perceptual quality.

Furthermore, considering the average performance across all mask ratios, our proposed algorithm demonstrates clear superiority. It achieves the best average PSNR (27.43 dB), the best average SSIM (0.898), and the lowest (best) average LPIPS (0.061) compared to all other methods evaluated on the Cityscapes dataset, underscoring its overall effectiveness and robust semantic guidance.

### 4.3. Ablation Studies on the Cityscapes Dataset

#### 4.3.1. Effectiveness of Semi-Supervised Semantic Feedback Recorrection

To investigate the contribution of our proposed semi-supervised semantic feedback recorrection module, we conduct an ablation study by removing it from our full pipeline. Our model architecture can be broadly conceptualized as a two-stage process: an initial coarse inpainting stage followed by a refinement stage. The semi-supervised semantic feedback recorrection module is designed to bridge these two stages, leveraging semantic segmentation guidance under a semi-supervised learning framework to enhance the intermediate coarse results before they are fed into the refinement network. By ablating this module, we aim to directly evaluate its effectiveness and the advantages conferred by integrating semantic segmentation priors through semi-supervised learning. Specifically, we will compare the quality of the inpainted results produced by the pipeline with and without this semantic correction step, focusing on the differences between the initial coarse outputs and the final refined outputs under both configurations.

Table 4 presents a quantitative evaluation demonstrating the significant impact of incorporating our semi-supervised semantic feedback recorrection module. The “Initial Inpainting” column presents results after the first stage (initial inpainting). The “Refined Inpainting” column shows the final outputs from our complete three-stage pipeline, which incorporates the initial inpainting, our semi-supervised semantic feedback recorrection module as the second stage, and a refined inpainting stage as the third stage. The data reveals that the full pipeline, yielding the refined inpainting results, achieves substantially superior performance over the initial inpainting baseline across all metrics (PSNR, SSIM, and LPIPS) on the Cityscapes dataset. This significant uplift underscores the collective efficacy of the semantic correction module and the subsequent refined inpainting stage. For instance, average PSNR improves from 24.98 dB (initial) to 27.43 dB (final), and average SSIM increases from 0.854 to 0.898. Concurrently, the perceptually oriented LPIPS score, where lower is better, reduces from an average of 0.116 to 0.061. These consistent improvements across various mask ratios, detailed in Table 4, point to the critical role of the subsequent enhancement process, with the semi-supervised semantic feedback recorrection being a key enabler.

The performance improvement from initial to final outputs, documented in Table 4, validates the effectiveness of the semi-supervised semantic feedback recorrection module. The overall performance gain stems from the collaboration between this module and the subsequent refined inpainting stage. Specifically, the recorrection module enhances semantic comprehension of coarsely inpainted regions and corrects inconsistencies to provide a more reliable semantic map. This optimized semantic foundation enables the refined inpainting stage to synthesize contextually appropriate details and textures more accurately. Therefore, incorporating semi-supervised semantic feedback recorrection is necessary for achieving this performance improvement and contributes to obtaining improved final inpainting results. The distinct advantages of this semi-supervised approach for semantic recorrection, particularly when compared against using a standard pre-trained semantic segmentation model to inform the refined inpainting stage, are further detailed and ablated in Section 4.3.2.

#### 4.3.2. Evaluating Semantic Segmentation Models for Enhanced Inpainting Guidance

Building upon the overall pipeline efficacy demonstrated in Section 4.3.1, this section specifically investigates the contribution of our semi-supervised semantic feedback recorrection module by evaluating the semantic segmentation model it employs. We aim to verify that our specialized segmentation model, refined through the processes detailed in Section 3.3, offers superior guidance for the subsequent refined inpainting stage compared to a conventionally pre-trained alternative. A key focus is its ability to mitigate semantic error propagation from the initial inpainting.

To isolate the impact of the segmentation model’s training strategy, we conduct an ablation study. We compare the final inpainting performance (output of Stage 3) when guided by two differently trained segmentation models, both sharing the same network architecture. The first, termed the baseline pre-trained model, is trained solely with supervised learning on ground-truth labels, without any exposure to the inpainting task or iterative refinement. This configuration corresponds to the “NO” condition in Table 5 and serves as a benchmark. The second is our proposed segmentation model, which is the optimally trained model resulting from our complete Stage 2 design, incorporating iterative refinement and adaptation to the inpainting task. This corresponds to the “YES” condition in Table 5. This experiment directly assesses the benefits of our model’s enhanced training and task-specific adaptations for guiding the refined inpainting process.

The quantitative results presented in Table 5 validate the effectiveness of our approach. Specifically, when the refined inpainting stage is guided by the baseline pre-trained model—which lacks iterative refinement and inpainting-aware adaptation—its capacity to correct semantic errors from the initial inpainting remains limited, potentially leading to error accumulation throughout the pipeline. In contrast, employing our specifically proposed segmentation model to guide the inpainting process substantially enhances the final restoration outcomes. This is evidenced by consistent performance improvements across all evaluation metrics on the Cityscapes dataset: the average PSNR increases from 26.41 dB to 27.43 dB, SSIM improves from 0.866 to 0.898, and the LPIPS score decreases markedly from 0.087 to 0.061. These findings establish that a semantic segmentation model tailored for the inpainting task and iteratively optimized contributes to enhanced restoration performance, mitigates the propagation of semantic errors, and yields more accurate and realistic results.

Furthermore, we monitored the performance evolution of the segmentation models within the feedback loop. Our analysis indicates that after the semi-supervised iterative optimization, both the final semantic segmentation model for original images and the final inpainting-aware model demonstrated enhanced segmentation capability. This ensures that they provide higher-quality semantic guidance for the refined inpainting stage, which directly corresponds to the comprehensive improvement in inpainting performance observed in Table 5.

#### 4.3.3. Qualitative Evaluation

**Figure 10:**
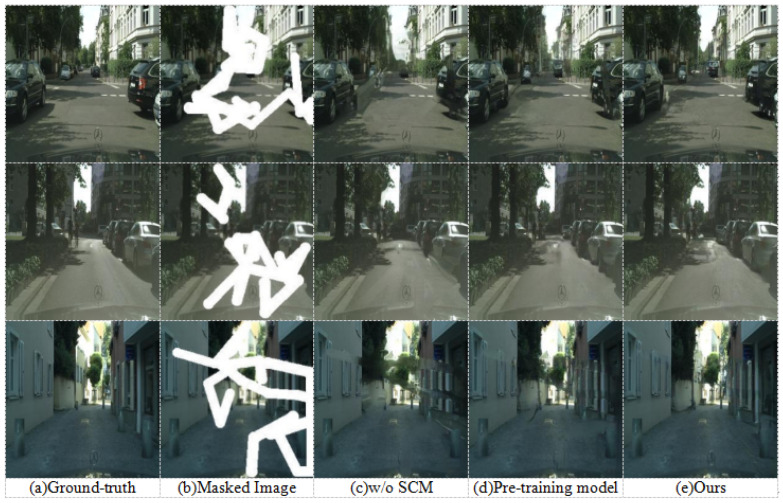
Qualitative comparison on Cityscapes dataset. The figure contains three rows of different scenes, with columns defined as (**a**) ground-truth, (**b**) masked image, (**c**) results from ablation without the semi-supervised semantic feedback recorrection module (“w/o SCM”), (**d**) results using the pre-trained segmentation model (“Pre-training model”), and (**e**) results from our method (“Ours”).

To visually assess the effectiveness of our proposed method and the contributions of its key components, this section presents a qualitative evaluation through ablation experiments. Random masks were applied to images from the Cityscapes dataset to facilitate this comparison. Figure 10 provides a visual comparison of the inpainting results. Each row contrasts the output of our method, labeled “Ours”, with two key ablation studies. The first, “w/o SCM”, represents the outcome when the semi-supervised semantic feedback recorrection module is removed. The second, “Pre-training model”, shows results from using the pre-trained segmentation model for guidance, a configuration consistent with the baseline discussed in Section 4.3.2. The original “Ground-truth” and “Masked Image” are also provided for context. A closer examination of the results in Figure 10 reveals distinct differences in performance among the compared methods, particularly in terms of semantic accuracy and the fidelity of repaired regions. Our proposed algorithm consistently demonstrates superior results, as will be detailed through specific examples below.

As shown in Figure 10, after removing the semi-supervised semantic segmentation and using the pre-trained model for segmentation, semantic-guided repair has indeed achieved some improvement at the semantic level. However, there remains a noticeable gap when compared to the overall repair performance of the proposed algorithm. In the third row of wall repair, after removing the semi-supervised semantic segmentation, the repair result exhibits texture blurring. When the pre-trained model is used for semantic segmentation, the error in the initial inpainting is amplified, resulting in significant semantic inconsistency and artifacts at the junction between the road and the wall.

In contrast, the images generated by our complete proposed algorithm consistently demonstrate superior quality. They exhibit clearer boundary definitions, improved structural coherence, and enhanced visual realism across various challenging scenarios. These visual results corroborate the quantitative findings, underscoring the critical role of our semi-supervised semantic feedback recorrection module with its specifically adapted and iteratively refined segmentation model in achieving high-fidelity, semantically coherent image inpainting.

### 4.4. Discussion on Failure Cases

While our algorithm demonstrates effective inpainting performance on the CelebA-HQ and Cityscapes datasets, its restoration quality markedly declines on the ADE20K dataset. This drop is primarily due to cross-image semantic consistency mechanism struggling with high intra-class diversity.

Our framework constructs category anchor vectors from labeled data to guide inpainting. For coherent categories like “human face”, this is effective. Yet, for broad categories in ADE20K (e.g., “building”, which includes Gothic buildings, modern apartments, and rural cottages), the anchor vector becomes overgeneralized. It fails to capture the distinct features of specific subclasses.

This overgeneralization causes a semantic mis-mapping during feedback recorrection. When the model processes a subclass not well represented in the anchor, it may map its features to a similar but semantically incorrect subclass. This semantic error leads to structural misalignments and unrealistic content in the final output.

This constraint reflects the challenge identified by Yang et al. [44], who pointed out that the problem of significant intra-class variability and data imbalance frequently compromises model robustness. Similarly, MMSeg [45], as a multi-task semi-supervised learning framework, underscores that datasets exhibiting heterogeneous semantic distributions often induce errors in pseudo-label generation. These observations substantiate the limitations of the proposed approach on the ADE20K dataset: when semantic categories exhibit substantial intra-class diversity, an over-reliance on cross-image semantic consistency fails to capture scene-specific subtle variations, resulting in a decline in task performance.

**Figure 11:**
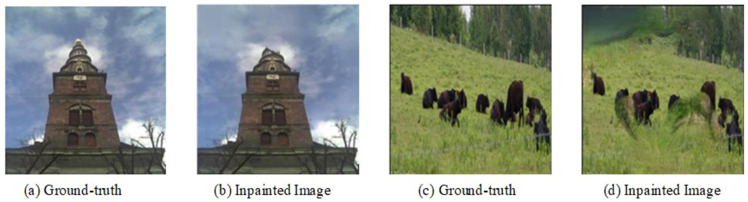
Typical failure cases of ADE20K dataset (Left: Building facade restoration. Right: Animal region inpainting in an outdoor scene).

Figure 11 illustrates typical failure modes of our algorithm on the challenging ADE20K dataset. For instance, in the building facade example (Figure 11a,b), when attempting to inpaint the damaged structure, the model erroneously incorporates textural features from the surrounding sky or vegetation. Similarly, in the outdoor scene featuring animals (Figure 11c,d), the algorithm struggles to preserve the animal’s form, instead generating irregular grassland-like textures within the animal region. These examples underscore a key limitation: the model’s cross-image semantic consistency mechanism can be misled by the significant intra-class visual diversity prevalent in ADE20K. Consequently, when the inherent semantic context of a target region is ambiguous or conflicts with features from visually similar but semantically different regions leveraged by the consistency mechanism, significant inpainting errors can occur.

In essence, these findings highlight that the effectiveness of our proposed semantic consistency mechanism is sensitive to the quality and nature of semantic annotations, particularly in datasets with high visual and semantic diversity like ADE20K. To mitigate such failure cases and further enhance performance, we need to develop more robust consistency constraints with reduced sensitivity to intra-class variance. This could involve exploring adaptive mechanisms that better discern when to rely on local context versus global semantic priors, particularly in complex scenes where semantic labels may not fully capture all nuanced visual attributes.

## 5. Conclusions

This research presents a novel semi-supervised semantic feedback recorrection algorithm for image inpainting. Our methodology integrates semantic segmentation with the inpainting process, which substantially improves the perceptual quality and semantic consistency of the reconstructed images. A core component is an interactive feedback mechanism; here, the initial inpainting output guides the semantic segmentation model. This iterative refinement allows the segmentation to correct initial inaccuracies before directing subsequent inpainting steps, leading to a marked improvement in the overall fidelity of the results. Extensive experimental validation demonstrates that this optimized semantic segmentation effectively rectifies semantic errors and significantly enhances the overall fidelity of the inpainted images. The semi-supervised learning strategy reduces reliance on labeled data, which enhances model robustness and minimizes annotation costs. The efficacy of the proposed interactive mechanism is further validated through comprehensive ablation analyses.

Nonetheless, we recognize certain limitations inherent to our approach that necessitate further research and optimization. Specifically, the labeling requirements for semantic maps within the dataset may constrain the algorithm’s generalizability, especially in scenarios involving broad category classifications, which could impair semantic correction accuracy. Moreover, the multi-iteration training paradigm and the utilization of multiple models result in increased training duration and elevated computational resource consumption. The proposed framework operates through three distinct phases: initial inpainting, semantic feedback recorrection, and refined inpainting. This architecture is inherently non-end-to-end, as its core semantic feedback recorrection module depends on a separately trained semi-supervised process involving pseudo-label generation and iterative model optimization. Consequently, the computational burden is not confined to a single forward pass but is significantly attributed to this independent training stage. This structure makes conventional metrics like FLOPs or parameter count insufficient for fully capturing the framework’s computational complexity. This design is a conscious trade-off, sacrificing simplicity for higher inpainting quality through staged, refined processing, particularly in correcting complex semantic deviations.

Future research will focus on two key directions: first, the exploration of more robust feature alignment and correction strategies to enhance model performance when processing data with high intra-class variance; second, the investigation of lightweight correction mechanisms and their integration into end-to-end training schemes, aiming to optimize both training and inference efficiency while maintaining high performance. Overall, this work provides an effective new paradigm for addressing semantically consistent restoration in complex scenes, offering significant theoretical insights and practical value for the field of image inpainting.

## Figures and Tables

**Table 1:** Comparison of real label images with different proportions on Cityscapes dataset.

Mask	PSNR/dB			SSIM↑			LPIPS↓		
	1/8	1/4	1/2	1/8	1/4	1/2	1/8	1/4	1/2
(0.0, 0.2]	30.23	31.58	**32.03**	0.936	0.948	**0.964**	0.068	0.039	**0.022**
(0.2, 0.4]	23.97	25.78	**26.95**	0.862	0.878	**0.899**	0.102	0.086	**0.063**
(0.4, 0.6]	21.48	22.96	**23.31**	0.781	0.813	**0.832**	0.147	0.112	**0.098**
Average	25.23	26.77	**27.43**	0.860	0.880	**0.898**	0.096	0.079	**0.061**

Note: ↑ higher is better, ↓ lower is better. Bold values indicate the better result.

**Table 2:** Quantitative comparison of different image inpainting methods on the CelebA-HQ dataset.

Metric	Mask	RFR	CTSDG	MMT	W-Net	LG-Net	MDTG	Ours
**PSNR/dB**↑	(0.0, 0.2]	30.89	31.92	32.08	32.32	33.22	33.61	**33.82**
	(0.2, 0.4]	25.91	27.49	27.22	27.00	27.87	27.88	**27.93**
	(0.4, 0.6]	23.77	25.15	25.12	24.43	**25.62**	25.46	25.51
	Average	26.86	28.19	28.14	27.92	28.85	28.98	**29.13**
**SSIM**↑	(0.0, 0.2]	0.947	0.956	0.958	0.958	0.962	0.966	**0.971**
	(0.2, 0.4]	0.870	0.907	0.904	0.903	0.907	0.913	**0.918**
	(0.4, 0.6]	0.804	0.859	0.849	0.842	0.857	**0.863**	0.860
	Average	0.874	0.907	0.904	0.902	0.909	0.914	**0.916**
**LPIPS**↓	(0.0, 0.2]	0.031	0.041	0.018	0.023	0.016	0.013	**0.012**
	(0.2, 0.4]	0.079	0.068	0.045	0.040	0.040	0.034	**0.032**
	(0.4, 0.6]	0.122	0.073	0.059	0.061	0.064	0.054	**0.053**
	Average	0.073	0.068	0.041	0.060	0.039	0.043	**0.032**

Note: ↑ higher is better, ↓ lower is better. Bold values indicate the better result.

**Table 3:** Quantitative comparison of different image inpainting methods on the Cityscapes dataset.

Metric	Mask	RFR	CTSDG	MMT	LG-Net	W-Net	MDTG	Ours
**PSNR/dB**↑	(0.0, 0.2]	31.11	30.34	31.12	31.38	31.04	31.68	**32.03**
	(0.2, 0.4]	25.79	26.07	25.89	26.17	25.49	26.61	**26.95**
	(0.4, 0.6]	23.33	**24.28**	23.15	23.69	22.45	23.44	23.31
	Average	26.74	26.90	26.83	27.08	26.33	27.24	**27.43**
**SSIM**↑	(0.0, 0.2]	0.942	0.941	0.947	0.946	0.949	0.950	**0.964**
	(0.2, 0.4]	0.860	0.882	0.873	0.867	0.875	0.884	**0.899**
	(0.4, 0.6]	0.780	0.822	0.798	0.791	0.791	0.817	**0.832**
	Average	0.861	0.882	0.873	0.868	0.872	0.884	**0.898**
**LPIPS**↓	(0.0, 0.2]	0.038	0.069	0.039	0.037	0.042	0.027	**0.022**
	(0.2, 0.4]	0.090	0.104	0.092	0.087	0.099	0.066	**0.063**
	(0.4, 0.6]	0.140	0.140	0.159	0.131	0.158	0.103	**0.098**
	Average	0.089	0.104	0.097	0.085	0.099	0.065	**0.061**

Note: ↑ higher is better, ↓ lower is better. Bold values indicate the better result.

**Table 4:** Quantitative evaluation of the semi-supervised semantic feedback recorrection module on Cityscapes dataset using different mask ratios.

Mask	PSNR/dB↑		SSIM↑		LPIPS↓	
	Initial Inpainting	Refined Inpainting	Initial Inpainting	Refined Inpainting	Initial Inpainting	Refined Inpainting
(0.0, 0.2]	29.96	**32.03**	0.930	**0.964**	0.075	**0.022**
(0.2, 0.4]	23.64	**26.95**	0.859	**0.899**	0.112	**0.063**
(0.4, 0.6]	21.35	**23.31**	0.773	**0.832**	0.161	**0.098**
Average	24.98	**27.43**	0.854	**0.898**	0.116	**0.061**

Note: ↑ higher is better, ↓ lower is better. Bold values indicate the better result.

**Table 5:** Impact of segmentation model training strategy on inpainting performance on the Cityscapes dataset.

Mask	PSNR/dB↑		SSIM↑		LPIPS↓	
	NO	YES	NO	YES	NO	YES
(0.0, 0.2]	30.87	**32.03**	0.944	**0.964**	0.034	**0.022**
(0.2, 0.4]	25.67	**26.95**	0.868	**0.899**	0.091	**0.063**
(0.4, 0.6]	22.69	**23.31**	0.787	**0.832**	0.137	**0.098**
Average	26.41	**27.43**	0.866	**0.898**	0.087	**0.061**

Note: “NO”: Inpainting guided by a baseline segmentation model; “YES”: Inpainting guided by our proposed segmentation model. ↑ higher is better, ↓ lower is better. Bold values indicate the better result.

## Data Availability

The original contributions presented in this study are included in the article. Further inquiries can be directed to the corresponding author on reasonable request.
